# Building a network of TP53 and IGHV testing reference centers across Spain: the Red53 initiative

**DOI:** 10.1007/s00277-020-04331-9

**Published:** 2021-01-06

**Authors:** Francesc Bosch, Blanca Navarro, Marta Crespo, Miguel Alcoceba, Julio Bravo Sánchez, Barbara Tazón, Alicia Serrano, María García Álvarez, Lydia González Serrano, Pablo Alonso-Torres, Miguel Villanueva, Cristina Loriente, Pau Abrisqueta, Manel Peiró, José Antonio García-Marco, Marcos González, María José Terol

**Affiliations:** 1 Servei d’Hematologia, Vall d’Hebron Hospital Universitari, Experimental Hematology, Vall d’Hebron Institute of Oncology (VHIO), Vall d’Hebron Barcelona Hospital Campus, Barcelona, Spain; 2 Department of Hematology, University Clinic Hospital of Valencia, INCLIVA Institute, Valencia, Spain; 3 Department of Hematology, University Hospital of Salamanca (HUS/IBSAL), CIBERONC and Center for Cancer Research-IBMCC (USAL-CSIC), Salamanca, Spain; 4 Department of Hematology, Hospital Universitario Puerta de Hierro-Majadahonda, IDIPHIM-Segovia de Arana, Madrid, Spain; 5 Janssen Pharmaceutical Companies of Johnson & Johnson, Madrid, Spain; 6 ESADE Business School, Institute for Healthcare Management, Universitat Ramon Llull, Barcelona, Spain

**Keywords:** CLL, IGHV mutational status, TP53 gene mutations, Laboratory network

## Abstract

Among the different biomarkers predicting response in chronic lymphocytic leukemia (CLL), the most influential parameters are the mutational status of the IGHV genes and the presence of *TP53* gene disruptions. Nevertheless, these important assessments are not readily available in most centers dealing with CLL patients. To provide this molecular testing across the country, the Spanish Cooperative Group on CLL (GELLC) established a network of four analytical reference centers. A total of 2153 samples from 256 centers were analyzed over a period of 30 months. In 9% of the patients, we found pathological mutations in the *TP53* gene, whereas 48.96% were classified as IGHV unmutated. Results of the satisfaction survey of the program showed a Net Promoter Score of 85.15. Building a national network for molecular testing in CLL allowed the CLL population a broad access to complex biomarkers analysis that should translate into a more accurate and informed therapeutic decision-making.

## Introduction

During the last 5 years, relevant advances have occurred in the dissection of the genomic, epigenomic, and transcriptomic landscape of chronic lymphocytic leukemia (CLL), the most frequent leukemia in adults [1]. These advances have been translated into further understanding of the prognostic and predictive value of the biological characteristics, particularly genetic lesions associated with chemoresistance (reviewed in [2]).

Among the different genetic lesions described in CLL, deletions of the 17p13.1 chromosomal region (del17p) are found at different frequencies depending on the clinical stage of the disease, ranging from 1% in monoclonal B cell lymphocytosis to 20–40% in chemorefractory cases [3, 4]. Del17p invariably encompasses the locus of the tumor suppressor gene *TP53*, and mutations of the second allele, usually located in the DNA-binding domain, are found in ~ 80% of cases with del17p. Dysregulation of *TP53* is classically linked to refractoriness to chemotherapy and associated with a shorter time to first treatment and overall survival [5–7]. More importantly, around 2.5–4.5% of patients requiring front-line therapy harbor *TP53* gene mutations without del17p [6, 8–10], supporting the notion that in CLL mutational analysis of *TP53* gene is complementary to the standard assessment of del17p by fluorescent in situ hybridization (FISH). Accordingly, since *TP53* inactivation is determinant in therapeutic decisions in CLL, ascertainment of its disruption is considered mandatory before the onset of any treatment [1].

The mutational status of the IGHV genes represents one of the most important prognostic biomarkers in CLL. Compared with IGHV-mutated (M) cases, CLLs with unmutated IGHV (UM) are characterized by the presence of high-risk genetic lesions, a propensity to undergo clonal evolution, an association with a shorter time to first treatment (TTFT), a shorter progression-free survival when treated with chemotherapy combinations, and poor OS [11–15]. All things considered, the analysis of the IGHV mutational status is highly recommended when assessing the prognosis and determining the therapeutic strategy of patients with CLL [1, 2].

Because of technical difficulties and the need for specifically trained personnel, assessments of mutations of IGHV and *TP53* genes are not widely available in the great majority of laboratories dealing with the diagnosis of CLL. This hurdle turned of particular relevance when it became well-established the notion that *TP53* dysfunctional cases should not be treated with chemoimmunotherapy, in contrast with what has been observed with novel treatments (reviewed in [2]).

The Spanish Group of CLL (GELLC), with the cooperation of Janssen, launched the TP53 network initiative (RED53), a multicenter task force aimed to facilitate the *TP53* and IGHV mutational analysis to the whole country in a due time. The purposes of this network were to provide the mutational analysis of *TP53* and IGHV genes to the Spanish centers diagnosing patients with CLL, and to educate hematologists on the need for performing a molecular assessment of CLL patients to guide the therapeutic strategies.

Herein, we report the methodology and general outcomes of building this initiative that enabled the analysis of more than 2000 CLL cases in 2 years, representing another instance of how a national networking can improve the quality of health management of our patients.

## Methods

### Eligibility of cohort participants

Patients were eligible for the detection of IGHV or *TP53* gene mutations when they were diagnosed with CLL and required front-line treatment according to the criteria defined by the IWCLL guidelines [1, 16]. Patients without the need for treatment or those who were previously treated were excluded. Centers from the GELLC that sent samples fulfilled an anonymized form containing the following information of the patient needed for the sample analysis: WBC, lymphocyte count, number of CD19+/CD5+, and lymphocytes. These forms were previously distributed to the centers willing to participate in the network. The program started with the *TP53* gene analysis followed by the IGHV testing 1 year later. This project was centrally approved and reviewed by the Ethics Committee from the University Hospital Vall d’Hebron.

### Reference centers and procedures

Four Spanish centers (Hospital Clínico, Valencia; Hospital Puerta de Hierro, Madrid; Hospital Universitario, Salamanca; and Hospital Vall d’Hebron, Barcelona) were designated for molecular testing. All the participating centers constituting the network were assigned to send samples to one of those four centers, in such a way that there was an even geographical distribution of centers assigned. These referral centers were certified for *TP53* and IGHV analysis according to the criteria determined by the European Initiative for CLL (ERIC) [8, 17].

Physicians willing to perform a genetic test had to collect 8 mL of whole blood in EDTA, obtain an anonymized registry, and send the tube the same day of collection via an express courier to the referral center, adding the form previously mentioned.

The target time for response of *TP53* mutational analysis was established in a period inferior to 14 calendar days, and inferior to 21 calendar days for IGHV mutational analysis.

A customer satisfaction survey was performed after 1.5 years of the initiative launching. The goal was to establish the level of service to facilitate benchmarking with a view of enhancing performance. The methodology included to answer an online questionnaire form provided to all the centers participating in the network. In a scale from 1 to 10, items considered were speed of the shipping, quickness of the results, quality of the provided materials and brochures, global satisfaction with the program, degree of recommendation to other professionals, and finally, the Net Promoter Score (NPS) measured according to the standard procedure [18]. Shipping and laboratory testing were supported by Janssen Pharmaceuticals, Spain.

### Laboratory methodology

Genomic DNA was extracted from whole blood in EDTA. For IGHV analysis, the clonotypic IGHV-IGHD-IGHJ gene rearrangement was amplified by multiplex PCR using IGHV-leader and IGHJ primers. After Sanger sequencing, the mutational status was determined following ERIC guidelines (Rosenquist et al., Leukemia 2017) [17]. For *TP53* mutational analysis, exons 4 to 10 were amplified by PCR followed by Sanger sequencing. Results, interpretation, and reporting were performed following ERIC guidelines [8].

## Results

### Enrollment of patients

**Fig. 1:**
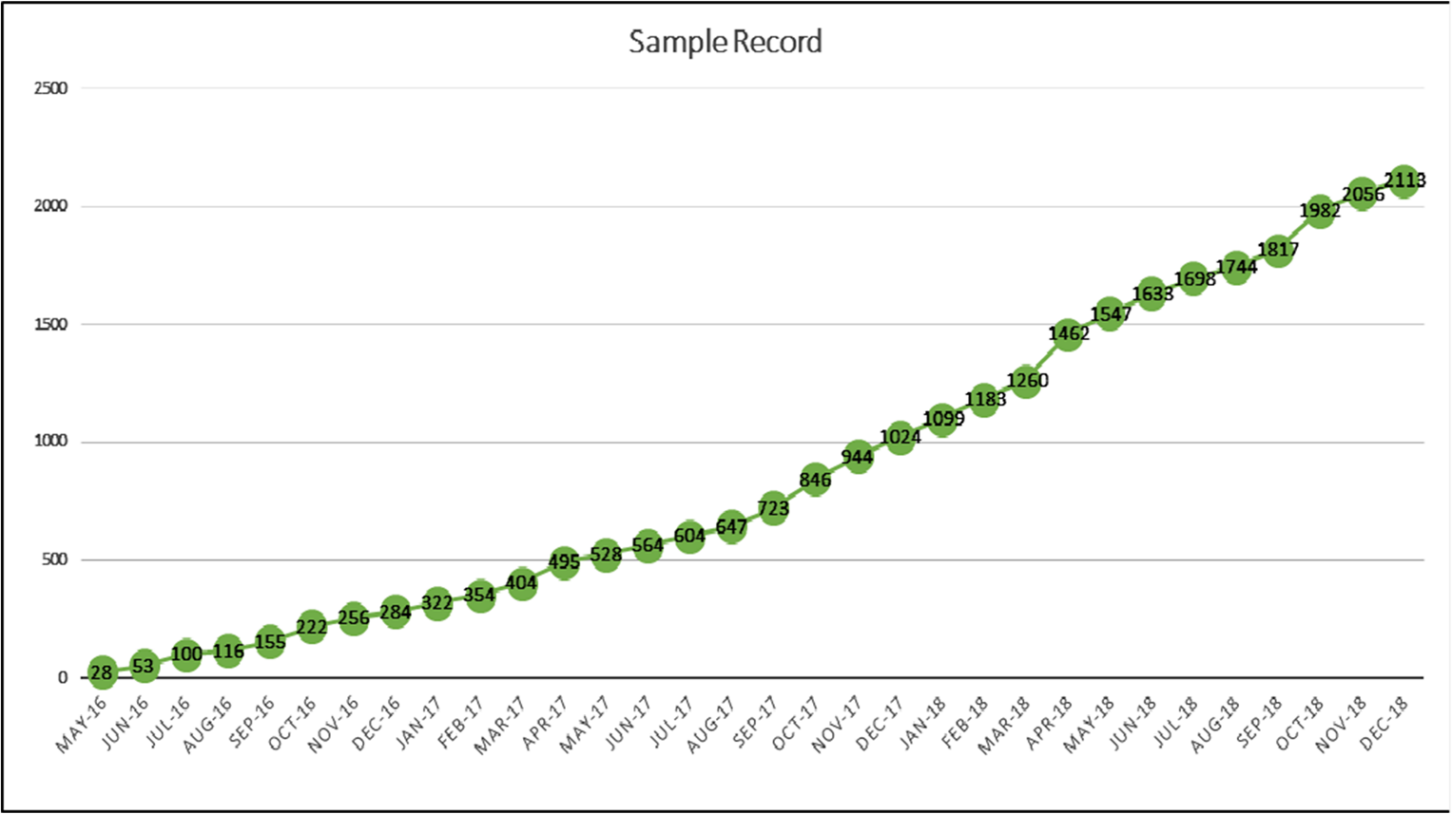
Number of included patients over time. Patients started to enter the RED53 program on May 2016, with a monthly rate of inclusions of 66 patients

From May 2016 to December 2018, a total of 2153 samples were analyzed from 256 institutions belonging to the Spanish Cooperative Group of CLL (GELLC) (Fig. 1). During the last year, when all the centers included in the study were already participating, the average number of inclusions was 88 patients per month (Fig. 1).

### Mutational analysis

Selected referral centers were certified for *TP53* and IGHV analysis according to the criteria determined by the European Initiative for CLL (ERIC) [8, 17]. In addition, centers shared samples to ascertain an absolute concordance on the result outputs. For these, samples from four patients with known TP53 somatic mutations were shared among the reference centers. Using the same protocol, all centers classified patients in the same IGHV category and were able to identify and report somatic mutations in TP53.

During this period of analysis, a total of 2153 samples were sent for *TP53* gene and/or IGHV mutational analysis. Altogether, 85 samples (3%) had not enough quality for molecular testing, including cases without PCR amplification or with low number of cells.

**Fig. 2:**
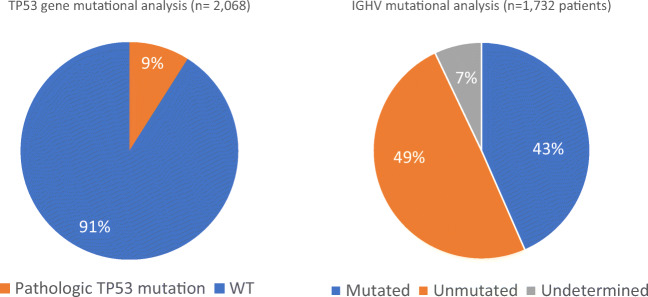
Chart distribution of the TP53 and IGHV gene mutational analysis. In 9% of patients, *TP53* gene disruptions were found, whereas 48.96% of the whole series expressed unmutated IGHV genes

*TP53* gene mutations were tested in 2068 cases. Among them, 182 (9%) patients were classified as having a pathological mutation, and the remaining 1886 patients (91%) were considered wild-type for *TP53* (Fig. 2).

As per the IGHV mutational analysis, a total of 1788 patients were analyzed. In 56 cases (3.1%), there was no amplification due to the low number of cells or low quality of samples. Among the remaining 1732 cases, 753 (43.48%) were classified as M-IGHV and the remaining 848 (48.96%) as having UM IGHV genes (Fig. 2). In 7.5% of the cases (*n* = 131), the result was considered undetermined because of polyclonal rearrangements (*n* = 34; 1.96%), unproductive (*n* = 27; 1.56%), or biclonal rearrangements with discordant mutational status (*n* = 70; 4.04%) were obtained.

Median turnaround time for *TP53* mutational status was 9.7 days (95% CI 9.4–9.9 days) which was inferior to the established target time (< 10 days) in 89% of the cases. For the IGHV mutational status analysis, median turnaround time for response was 13 days (95% CI 12.9–14.1 days), again being inferior to the targeted time (< 21 days) in 91% of the cases.

### Satisfaction survey

A total of 128 centers (50%) answered the satisfaction survey. Overall assessment of the program was considered excellent. Thus, with a scale from 1 to 10 (being 10 the best), the speed of the shipping was rated 9; quickness on delivering results, 8.65; quality and contents of informative materials and brochures, 9.1 and 9.07, respectively; adhesion to the program, 9.3; and overall satisfaction, 9.14. Finally, the NPS of the project was quantified as 85.15.

## Discussion

The most relevant biomarkers in guiding treatment decisions in CLL are the mutational status of IGHV genes and the disruptions of *TP53* gene (reviewed in [2]). Unfortunately, in clinical practice limited laboratory capacity is, in general, a bottleneck for these determinations as they require a degree of complexity not always existing in the great majority of centers dealing with patients diagnosed with CLL. To make these determinations accessible to all the patients in the country, the GELLC designed a network of referral centers for IGHV and *TP53* mutational testing for CLL patients requiring front-line treatment.

Herein, we present the results obtained during 2 years since the beginning of the network. Only 3.1% of samples arrived to the referral centers in bad condition, which seems acceptable, given the big number of participating centers spread around the country. The results found were consistent with the ones observed in previously reported series of patients having an advanced disease, with a higher percentage of UM IGHV over mutated, and around 10% of cases with pathological gene mutations of *TP53*. These figures are also resembling the ones observed in front-line clinical trials with chemoimmunotherapy or target treatments [14, 19, 20]. As per IGHV mutations, 7% of cases were considered undetermined. These figures differ from what it was previously reported by ERIC consortium, as they found 11.5% difficult cases and 1.7% cases classified as undetermined [17, 21]. Such differences that could be explained by a variety of reasons: first, at least 30% cases were analyzed using only genomic DNA; second, some of the undetermined cases had not enough tumoral lymphocytes (i.e., small lymphocytic lymphomas). The network will solve part of the undetermined by promoting the use of cDNA in problematic cases and requiring a minimum of 5000 × 10^9^/L malignant lymphocytes to process the sample.

The establishment of this national RED53 network provided several benefits for both patients and physicians, as reflected by the high score observed in the satisfaction survey. First, it facilitated the access to test these important predictive factors to almost all patients requiring therapy. Second, the program educated physicians on the need for assessing molecular biomarkers in patients diagnosed with CLL in need of treatment. Third, the program allowed to build a biobank with part of the samples that should be the basis for future translational research initiatives. Finally, the program enables expertise to be shared and synergies to be brought into play.

A network is an agreement among multiple organizations to address problems which could not be resolved by individual organizations [22]. Networking has become more significant in the health sector for the purpose of advancing the field of science, covering health needs, and prioritizing the allocation of health resources [23]. Laboratory networks are vital to well-functioning public health systems for disease study and control efforts [24–26]. As RED53 network organized activities among different groups to increase efficiency, it could be considered a coordinative network [27]. The network presented here served as platform for increasing the quality of care for patients diagnosed with CLL and provided another example of the way scientific initiatives should be designed. The results obtained are consistent with the description of good performance in a collaborative relationship, based on the effectiveness, support, integral character of the solution, and robustness [28]. Such a good performance is based on trust [28], which relay on previously shared experiences within the GELLC and by the professional reputation of the four centers in charge of the tests. Finally, this network supports the partnership between disease cooperative groups and pharmaceutical companies, and brings clinical practice closer to precision medicine. It can be considered a successful example of public-private partnership due to the nature of the participants and the results provided, which, as identified by several authors, include a better value for money and a reduction of some pressure on public budgets, allowing at the same time for a greater innovation [29].

In conclusion, this coordinative network for molecular testing allowed for an increased quality of care for patients with a cancer diagnosis. We conceive that this model could be translated to other countries or cooperative groups for the benefit of patients with momentous diseases.
